# Acceptability of healthcare interventions: an overview of reviews and development of a theoretical framework

**DOI:** 10.1186/s12913-017-2031-8

**Published:** 2017-01-26

**Authors:** Mandeep Sekhon, Martin Cartwright, Jill J. Francis

**Affiliations:** City, University of London, Northampton Square, London, EC1V 0JB UK

**Keywords:** Acceptability, Defining constructs, Theory development, Complex intervention, Healthcare intervention

## Abstract

**Background:**

It is increasingly acknowledged that ‘acceptability’ should be considered when designing, evaluating and implementing healthcare interventions. However, the published literature offers little guidance on how to define or assess acceptability. The purpose of this study was to develop a multi-construct theoretical framework of acceptability of healthcare interventions that can be applied to assess prospective (i.e. anticipated) and retrospective (i.e. experienced) acceptability from the perspective of intervention delivers and recipients.

**Methods:**

Two methods were used to select the component constructs of acceptability. 1) An overview of reviews was conducted to identify systematic reviews that claim to define, theorise or measure acceptability of healthcare interventions. 2) Principles of inductive and deductive reasoning were applied to theorise the concept of acceptability and develop a theoretical framework. Steps included (1) defining acceptability; (2) describing its properties and scope and (3) identifying component constructs and empirical indicators.

**Results:**

From the 43 reviews included in the overview, none explicitly theorised or defined acceptability. Measures used to assess acceptability focused on behaviour (e.g. dropout rates) (23 reviews), affect (i.e. feelings) (5 reviews), cognition (i.e. perceptions) (7 reviews) or a combination of these (8 reviews).

From the methods described above we propose a definition: Acceptability is a multi-faceted construct that reflects the extent to which people delivering or receiving a healthcare intervention consider it to be appropriate, based on anticipated or experienced cognitive and emotional responses to the intervention. The theoretical framework of acceptability (TFA) consists of seven component constructs: affective attitude, burden, perceived effectiveness, ethicality, intervention coherence, opportunity costs, and self-efficacy.

**Conclusion:**

Despite frequent claims that healthcare interventions have assessed acceptability, it is evident that acceptability research could be more robust. The proposed definition of acceptability and the TFA can inform assessment tools and evaluations of the acceptability of new or existing interventions.

**Electronic supplementary material:**

The online version of this article (doi:10.1186/s12913-017-2031-8) contains supplementary material, which is available to authorized users.

## Background

Acceptability has become a key consideration in the design, evaluation and implementation of healthcare interventions. Many healthcare interventions are complex in nature; for example, they can consist of several interacting components, or may be delivered at different levels within a healthcare organisation [1]. Intervention developers are faced with the challenge of designing effective healthcare interventions to guarantee the best clinical outcomes achievable with the resources available [2, 3]. Acceptability is a necessary but not sufficient condition for effectiveness of an intervention. Successful implementation depends on the acceptability of the intervention to both intervention deliverers (e.g. patients, researchers or healthcare professionals) and recipients (e.g. patients or healthcare professionals) [4, 5]. From the patient’s perspective, the content, context and quality of care received may all have implications for acceptability. If an intervention is considered acceptable, patients are more likely to adhere to treatment recommendations and to benefit from improved clinical outcomes [6, 7]. From the perspective of healthcare professionals, if the delivery of a particular intervention to patients is considered to have low acceptability, the intervention may not be delivered as intended (by intervention designers), which may have an impact on the overall effectiveness of the intervention [8, 9].

In the United Kingdom, the Medical Research Council (MRC) has published three guidance documents for researchers and research funders in relation to appropriate methods for designing and evaluating complex interventions [10–12]. The number of references to acceptability has increased with each guidance publication which reflects the growing importance of this construct. The 2000 MRC guidance document makes no reference to acceptability, whereas the 2015 guidance refers to acceptability 14 times but lacks a definition and fails to provide clear instructions on how to assess acceptability.

The 2015 guidance focuses on conducting process evaluations of complex interventions. It offers examples of how patients’ acceptability may be assessed quantitatively, by administering measures of acceptability or satisfaction, and qualitatively, by asking probing questions focused on understanding how they are interacting with the intervention [12]. Nevertheless, it fails to offer a definition of acceptability or specific materials for operationalising it. Without a shared understanding of what acceptability refers to it is unclear how intervention developers are to assess acceptability for those receiving and delivering healthcare interventions.

### Attempts to define acceptability

Defining acceptability is not a straightforward matter. Definitions within the healthcare literature vary considerably highlighting the ambiguity of the concept. Specific examples of definitions include the terms ‘treatment acceptability’ [13–15] and ‘social acceptability’ [16–18]. These terms indicate that acceptability can be considered from an individual perspective but may also reflect a more collectively shared judgement about the nature of an intervention.

Stainszewska and colleagues (2010) argue that social acceptability refers to *“patients’ assessment of the acceptability, suitability, adequacy or effectiveness of care and treatment”* ([18], p.312). However, this definition is partly circular as it states that social acceptability entails acceptability. These authors also omit any guidance on how to measure patients’ assessment of care and treatment.

Sidani et al., (2009) propose that treatment acceptability is dependent on patients’ attitude towards treatment options and their judgement of perceived acceptability prior to participating in an intervention. Factors that influence patients’ perceived acceptability include the intervention’s “*appropriateness in addressing the clinical problem, suitability to individual life style, convenience and effectiveness in managing the clinical problem”* ([14], p.421). Whilst this conceptualisation of treatment acceptability can account for patients’ decisions in terms of wishing to complete treatments and willingness to participate in an intervention, it implies a static evaluation of acceptability. Others argue that perceptions of acceptability may change with actual experience of the intervention [19]. For example, the process of participating in an intervention, the content of the intervention, and the perceived or actual effectiveness of the intervention, are likely to influence patients’ perceptions of acceptability.

### Theorising acceptability

The inconsistency in defining concepts can impede the development of valid assessment instruments [20]. Theorising the concept of acceptability would provide the foundations needed to develop assessment tools of acceptability.

Within the disciplines of health psychology, health services research and implementation science the application of theory is recognised as enhancing the development, evaluation and implementation of complex interventions [10, 11, 21–25]. Rimer and Glanz (2005) explain “a theory presents a systematic way of understanding events or situations. It is a set of concepts, definitions, and propositions that explain or predict these events or situations by illustrating the relationship between variables” ([26] p.4).

We argue that theorising the construct of acceptability will lead to a better understanding of: (1) what acceptability is (or is proposed to be) (specifically whether acceptability is a unitary or multi-component construct); (2) if acceptability is a multi-component construct, what its components are (or are proposed to be); (3) how acceptability as a construct is proposed to relate to other factors, such as intervention engagement or adherence; and (4) how it can be measured.

### Aims and objectives

The aim of this article is to describe the inductive (empirical) and deductive (theoretical) methods applied to develop a comprehensive theoretical framework of acceptability. This is presented in two sequential studies. The objective of the first study was to review current practice and complete an overview of systematic reviews identifying how the acceptability of healthcare interventions has been defined, operationalised and theorised. The objective of the second study was to supplement evidence from study 1 with a deductive approach to propose component constructs in the theoretical framework of acceptability.

## Methods

### Study 1: Overview of reviews

Preliminary scoping searches identified no existing systematic review focused solely on the acceptability of healthcare interventions. However, systematic reviews were identified which considered the acceptability of healthcare and non-healthcare interventions alongside other factors such as effectiveness [27] efficacy [28] and tolerability [29]. We therefore decided to conduct an overview of systematic reviews of healthcare interventions that have included a focus on acceptability, alongside other factors (e.g. effectiveness, feasibility).

#### Search strategy

Systematic Reviews published from May 2000 (the 2000 MRC guidance was published in April 2000) to February 2016 were retrieved through a single systematic literature search conducted in two phases (i.e. the initial phase 1 search was conducted in February 2014 and this was updated in phase 2 February 2016). There were two search strategies applied to both phase 1 and phase 2 searches. The first strategy was applied to the Cochrane Database of Systematic Reviews (CDSR), based on the appearance of the truncated term “acceptab*” in article titles. The second search involved applying the relevant systematic review filter (Additional file 1) to the search engines OVID (Medline, Embase) and EBSCO Host (PsycINFO), and combining the review filter with the appearance of the term “acceptab*” in article titles. By searching for “acceptab*” within the article title only (rather than within the abstract or text), we also ensured that only reviews focused on acceptability as a key variable would be identified. Only reviews published in English were included as the research question specifically considered the word “acceptability”; this word may have different shades of meaning when translated into other languages, which may in turn affect the definition and measurement issues under investigation.

#### Screening of citations

Duplicates were removed in Endnote. All abstracts were reviewed by a single researcher (MS) against the inclusion and exclusion criteria (Table 1). To assess reliability of the screening process, another researcher (MC) independently reviewed 10% of the abstracts. There was 100% agreement on the abstracts included for full text review.

**Table 1:** Inclusion and exclusion criteria for the overview of reviews

Inclusion criteria	Exclusion criteria
All systematic reviews (including critical synthesis reviews) of a healthcare intervention A systematic review was defined as “a review of a clearly formulated question that uses systematic and explicit methods to identify, select and critically appraise relevant research and to collect and analyse data from the studies that are included in the review” (Moher et al., 2009, p.1) [64] Participant samples included all recipients and deliverers of healthcare interventions	Non-English systematic reviews Systematic reviews which only made reference to cost-effectiveness acceptability curves

#### Full text review and data extraction

One researcher (MS) retrieved all full text papers that met the inclusion criteria and extracted data using an extraction form. Two additional researchers (JF and MC) independently reviewed 10% of the included systematic reviews. The researchers extracted information on how acceptability had been defined, whether acceptability had been theorised, and when and how acceptability had been assessed. There were no disagreements in data extraction.

#### Assessment of quality

No quality assessment tool was applied as it is possible that poor quality systematic reviews would include information relevant to addressing the study aims and objectives.

#### Definitions of acceptability: consensus group exercises

To identify how acceptability has been defined one researcher (MS) extracted definitions from each of the systematic reviews. Where definitions of acceptability were unclear, a reasonable level of inference was used in order to identify an implicit definition where review authors imply their understanding of acceptability whilst not directly proposing a definition of acceptability (see results section for example of inferences).

To check reliability of the coding of extracted text reflecting implicit or explicit definitions seven research psychologists (including the three authors) were asked to classify the extracted text into the following categories: (1) Conceptual Definition (i.e. an abstract statement of what acceptability is); (2) Operational Definition (i.e. a concrete statement of how acceptability is measured); (3) Uncertain; and (4) No Definition. The consensus group was allowed to select one or more options that they considered applicable to each definition. All definitions from the included systematic review papers were extracted, tabulated and presented to the group, together with definitions of “conceptual” and “operational”. Explanations of these categories are presented in Table 2. One researcher (MS) facilitated a short discussion at the beginning of the task to ensure participants understood the “conceptual” and “operational” definitions. The review authors subsequently repeated the same exercise for extracted definitions from the updated phase 2 search.

**Table 2:** Definitions of key terms applied in theory development

Key term	Definition
Conceptual definition	Defines a construct in abstract or theoretical terms
Operational definition	Defines a construct by specifying the procedures used to measure that construct
Concept	Mental representation of a kind or category of items or ideas (APA, 2017) [65]
Construct	The building block for theorising (Glanz et al., 2008) [66]
Conceptualisation	Involves concept formation, which establishes the meaning of a construct by elaborating the nomological network and defining important subdomains of its meaning (p. 4 Hox 1997 [33])
Operationalization	Involves the translation of a theoretical construct into observable variables by specifying empirical indicators for the concept and its subdomains (p. 4 Hox, 1997 [33])

#### Synthesis

No quantitative synthesis was conducted. All extracted data were analysed by applying the thematic synthesis approach [30].

### Study 2: Development of a theoretical framework of acceptability

The methods applied to develop theory are not always described systematically in the healthcare and psychology literature [31]. Broadly, the most common approaches are data driven (bottom up/ inductive) and theory driven (top down/ deductive) processes [32–34]. The data driven process focuses on observations from empirical data to form theory, whereas the theory driven process works on the premise of applying existing theory in an effort to understand data. The process of theorising is enhanced when inductive and deductive processes are combined [35, 36]. To theorise the concept of acceptability, we applied both inductive and deductive processes by taking a similar approach described by Hox [33].

Hox proposed that, in order to theorise, researchers must (1) decide on the concept for measurement; (2) define the concept; (3) describe the properties and scope of the concept (and how it differs from other concepts); and (4) identify the empirical indicators and subdomains (i.e. constructs) of the concept. We describe below how steps 1-4 were applied in developing a theoretical framework of acceptability.

#### Step 1: Concept for measurement

We first agreed on the limits of the construct to be theorised: acceptability of healthcare interventions.

#### Step 2: Defining the concept

To define the concept of acceptability we reviewed the results of the overview of reviews, specifically the conceptual and operational definitions identified by both consensus group exercises and the variables reported in the behavioural and self-report measures (identified from the included systematic reviews). Qualitatively synthesising these definitions, we proposed the following conceptual definition of acceptability:

A multi-faceted construct that reflects the extent to which people delivering or receiving a healthcare intervention consider it to be appropriate, based on anticipated or experienced cognitive and emotional responses to the intervention.

This definition incorporates the component constructs of acceptability (cognitive and emotional responses) and also provides a hypothesis (cognitive and emotional responses are likely to influence behavioural engagement with the intervention). This working definition of acceptability can be operationalised for the purpose of measurement.

#### Step 3: Describing the properties and scope of the concept

Based on the conceptual definition we identified the properties and scope of the construct of acceptability using inductive and deductive methods to determine which constructs best represented the core empirical indicators of acceptability.

##### Inductive methods

**Fig. 1:**
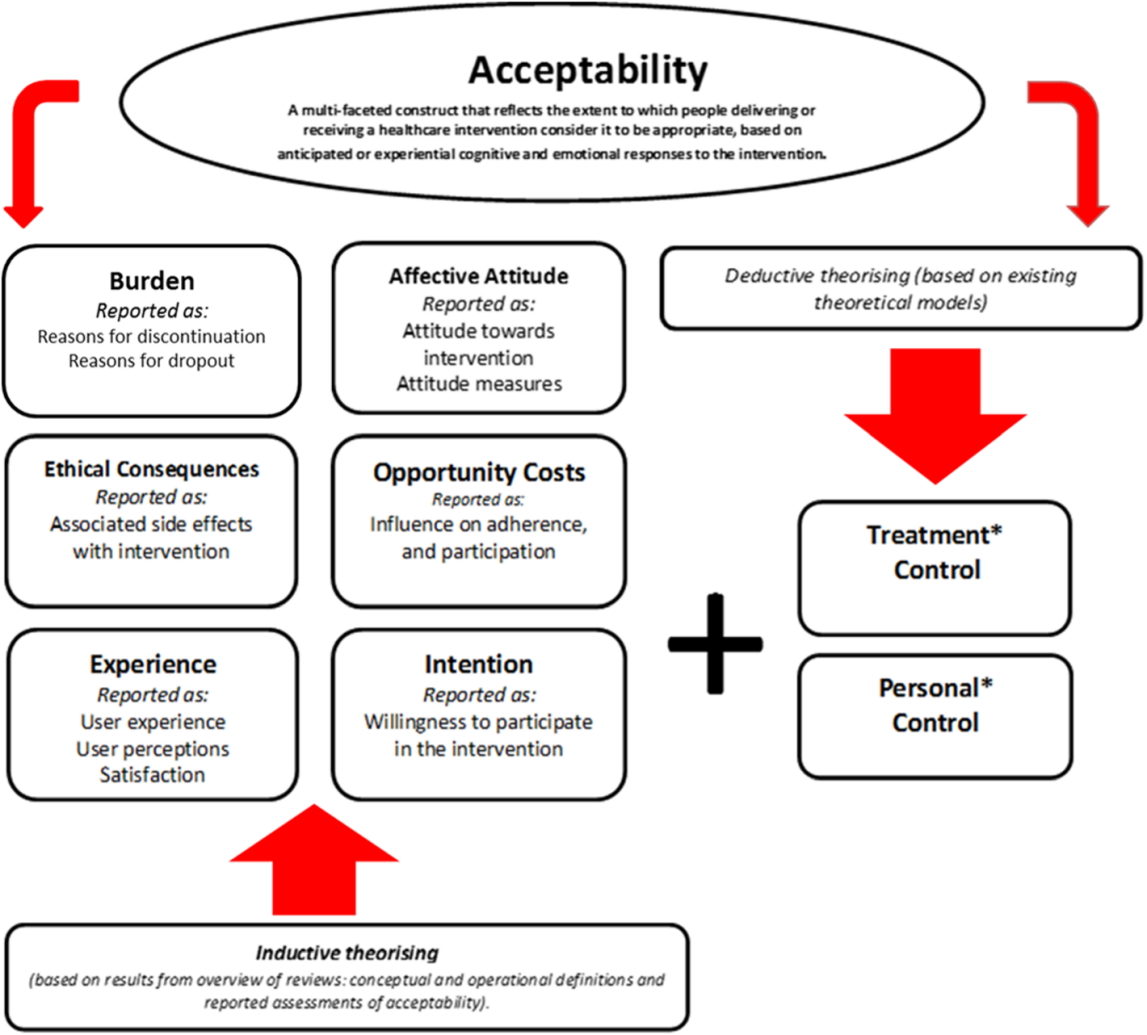
The theoretical framework of acceptability (v1). Note: In bold font are the labels we assigned to represent the examples of the variables applied to operationalise and assess acceptability based on the results from the overview (italic font). Note* Addition of the two control constructs emerging deductively from existing theoretical models

The application of inductive methods involved reviewing the empirical data that emerged from the overview of reviews. First, variables identified in the consensus group task to define acceptability, and the variables reported in the observed behavioural measures and self-report measures of acceptability, were grouped together according to similarity. Next, we considered what construct label best described each of the variable groupings. For example, the variables of “attitudinal measures”, and “attitudes towards the intervention (how patients felt about the intervention)” was assigned the construct label “affective attitude”. Figure 1 presents our conceptual definition and component constructs of acceptability, offering examples of the variables they incorporate. This forms our preliminary theoretical framework of acceptability, TFA (v1).

##### Deductive methods

The deductive process was conducted iteratively using the following three steps:

(1) We considered whether the coverage of the preliminary TFA (v1) could usefully be extended by reviewing the identified component constructs of acceptability against our conceptual definition of acceptability and the results of the overview of reviews.

(2) We considered a range of theories and frameworks from the health psychology and behaviour change literatures that have been applied to predict, explain or change health related behaviour.

(3) We reviewed the constructs from these theories and frameworks for their applicability to the TFA. Examples of theories and frameworks discussed include the Theory of Planned Behaviour (TPB) [37] (e.g. the construct of Perceived Behavioural Control) and the Theoretical Domains Framework (TDF) [38] (e.g. the constructs within the Beliefs About Capabilities domain). We discussed whether including additional constructs would add value to the framework in assessing acceptability, specifically if the additional constructs could be measured as cognitive and / or emotional responses to the intervention. The TPB and the TDF focus on beliefs about performing a behaviour whereas the TFA reflects a broader set of beliefs about the value of a healthcare intervention. We concluded that there was a more relevant theory that provides better fit with the TFA, the Common Sense Model (CSM) of self-regulation of health and illness [37]. The CSM focuses on beliefs about a health threat and coping procedures that might control the threat. This approach is thus consistent with the focus of the TFA on acceptability of healthcare interventions. The CSM proposes that, in response to a perceived health threat, individuals spontaneously generate five kinds of cognitive representation of the illness based around identity (i.e. associated symptoms), timeline, cause, control/cure, and consequences. Moss-Morris and colleagues [38] distinguished between personal control (i.e. the extent to which an individual perceives one is able to control one’s symptoms or cure the disease) and treatment control (i.e. the extent to which the individual believes the treatment will be effective in curing the illness). The third step in the deductive process resulted in the inclusion of both treatment control and personal control as additional constructs within the TFA (v1) (Fig. 1). With these additions the framework appeared to include a parsimonious set of constructs that provided good coverage of acceptability as defined.

#### Step 4: Identifying the empirical indicators for the concept’s constructs

Having identified the component constructs of acceptability, we identified or wrote formal operational definitions for each of the constructs within the TFA (v1). This was done to check that the constructs were conceptually distinctive. We first searched the psychological literature for definitions. If a clear definition for a construct was not available in the psychological literature, standard English language dictionaries and other relevant disciplines (e.g. health economic literature for a definition of “opportunity costs”) were searched. For each construct, a minimum of two definitions were identified. Extracted definitions for the component constructs were required to be adaptable to refer directly to “the intervention” (see results section for examples). This process resulted in revisions to the TFA (v1) and the development of the revised TFA (v2).

## Results

### Study 1: Overview of reviews

#### Characteristics of included reviews

**Fig. 2:**
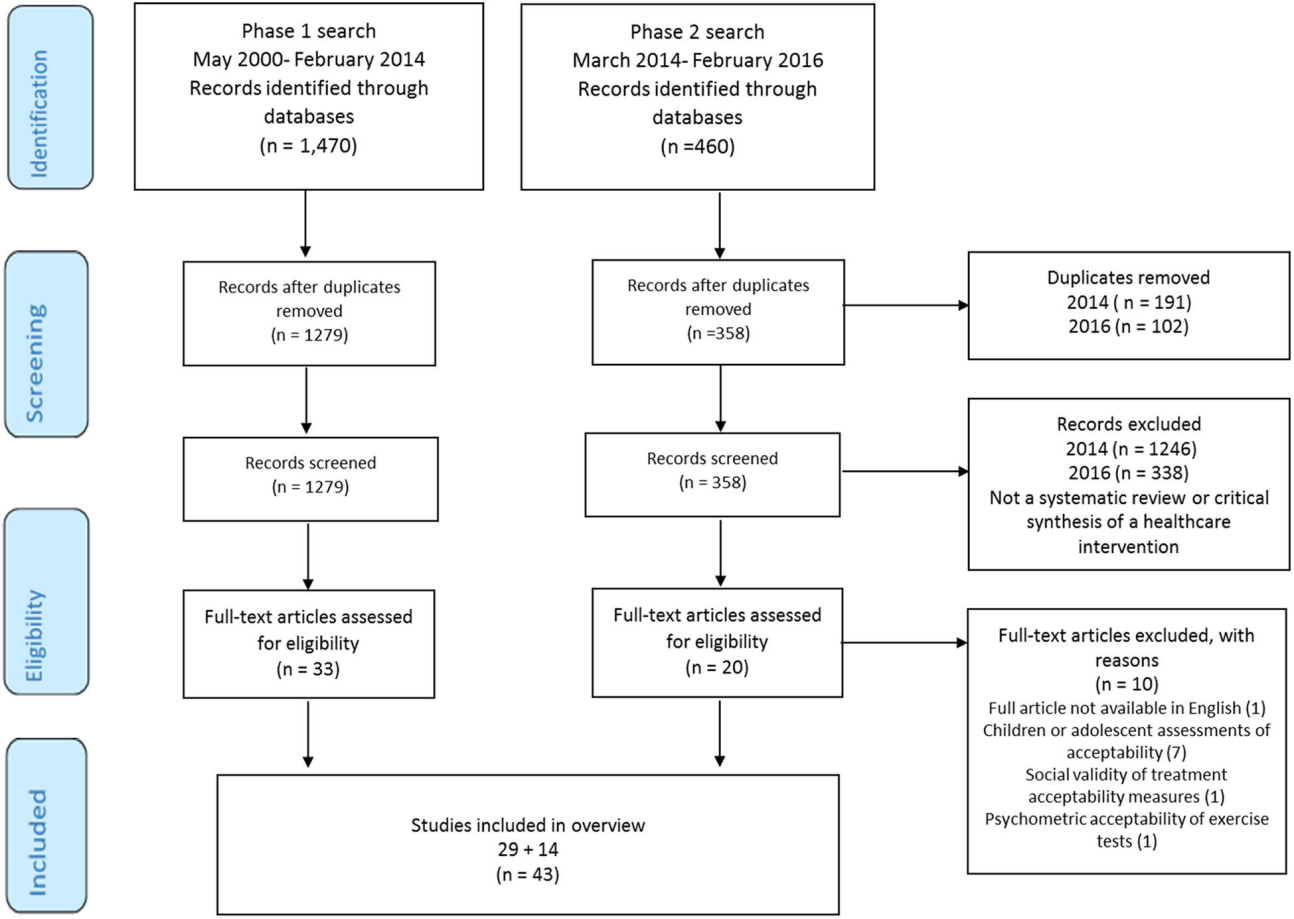
PRISMA diagram of included papers for searches completed in February 2014 and 2016

The databases searches identified 1930 references, with 1637 remaining after de-duplication. After screening titles and abstracts, 53 full texts were retrieved for further examination. Of these, ten articles were excluded for the following reasons: seven articles focused on children’s and adolescents’ acceptability of the intervention, one could not be obtained in English, one article focused on social validity of treatment measures in education psychology, and one article focused on the psychometric properties of exercise tests. Thus, a total of 43 publications were included in this overview (Additional file 2). The breakdown of the search process for phase 1 and phase 2 is represented in Fig. 2.

#### Assessment of quality

The methodological quality of individual studies was assessed in 29 (67%) of the 43 reviews. The Cochrane Tool of Quality Assessment was applied most frequently [39] (18 reviews: 62%). Other assessments tools applied included the Jadad Scale [40] (three reviews: 10%), the Critical Appraisal Skills Programme (CASP) guidelines [41] (three reviews: 10%), CONSORT guidelines [41] (two reviews: 6%); Grade scale [42] (one review: 3%), Effective Public Health Practice Project (EPHPP) quality assessment tool [43] (one review: 3%) and United States Preventive Services Task Force grading system [44] (one review: 3%).

#### Assessment of acceptability

Twenty-three (55%) reviews assessed acceptability using various objective measures of behaviour as indicators of acceptability: dropout rates, all-cause discontinuation, reason for discontinuation and withdrawal rates (Additional file 3). Twelve (26%) of the reviews reported that they assessed acceptability using self-report measures, which included responses to hypothetical scenarios, satisfaction measures, attitudinal measures, reports of individuals on their perceptions of, and experiences with, the intervention, and opened-ended interview questions (Additional file 4). None of the reviews specified a threshold criterion, i.e., the number of participants that needed to withdraw /discontinue treatment, for the intervention to be considered unacceptable.

Eight (19%) reviews assessed acceptability using both objective measures of behaviour and self-reported measures. These included two reviews measuring adherence and satisfaction [45, 46], three reviews focusing on dropout rates, take-up rates, reasons for discontinuation and a satisfaction measure [47–49] one review combining the time taken for wound healing alongside a measure of satisfaction and comfort [29], and two reviews using semi-structured interviews to explore participant experience of the intervention alongside intervention take-up rates [50, 51].

We also extracted data on the time at which studies in each of the reviews assessed acceptability relative to the delivery of the intervention (Additional file 5). Two of the reviews (5%) assessed acceptability pre-intervention, which involved participants agreeing to take part in screening for a brief alcohol intervention [52] and willingness to participate in HIV self–testing [53]. Seven (16%) of the reviews assessed acceptability during the intervention delivery period, while 17 (40%) assessed acceptability post-intervention. Fourteen reviews (33%) did not report when acceptability was measured, and in three (7%) of the reviews it was unclear when acceptability was measured. Within these three reviews, it was unclear whether interpretations of intervention acceptability were based on anticipated (i.e. prospective) acceptability or experienced (i.e. concurrent or retrospective) acceptability.

#### Use of theory

There was no mention of theory in relation to acceptability in any of these 43 reviews. None of the review authors proposed any link between their definitions (when present) and assessments of acceptability and existing theory or theoretical models (i.e. scientific and citable theories/models). Moreover, none of the reviews proposed any link between implicit theories and their definitions and assessments of acceptability, or theory emerging during the studies reported in the systematic reviews. No links were proposed because, by definition, an implicit theory is not articulated.

#### Definitions of acceptability: consensus group exercise

Extracted definitions of acceptability required a minimum of four of seven judges to endorse it as representing either an operational or conceptual definition. From the 29 extracts of text (phase 1 search results), the expert group identified 17 of the extracts as being operational definitions. Operational definitions included measureable factors such as dropout rates, all cause discontinuation, treatment discontinuation and measures of satisfaction. Some reviews indicated that acceptability was measured according to a number of indicators, such as effectiveness and side effects. The remaining 12 extracted definitions were not reliably classified as either operational or conceptual and were disregarded. For the 14 extracted definitions based on the phase 2 search results, two endorsements (from three judges) was required for a definition to be considered as operational or conceptual. Seven definitions were considered operational definitions of acceptability, three definitions were identified as conceptual and four extracts were not reliably classified as either. Conceptual definitions included: “acceptability, or how the recipients of (or those delivering the intervention) perceive and react to it” ([49] p. 2) “…patients reported being more willing to be involved” ([54] p. 2535) and “women were asked if they were well satisfied, unsatisfied or indifferent or had no response” with the intervention ([55] p. 504).

### Study 2: Theoretical framework of acceptability

**Fig. 3:**
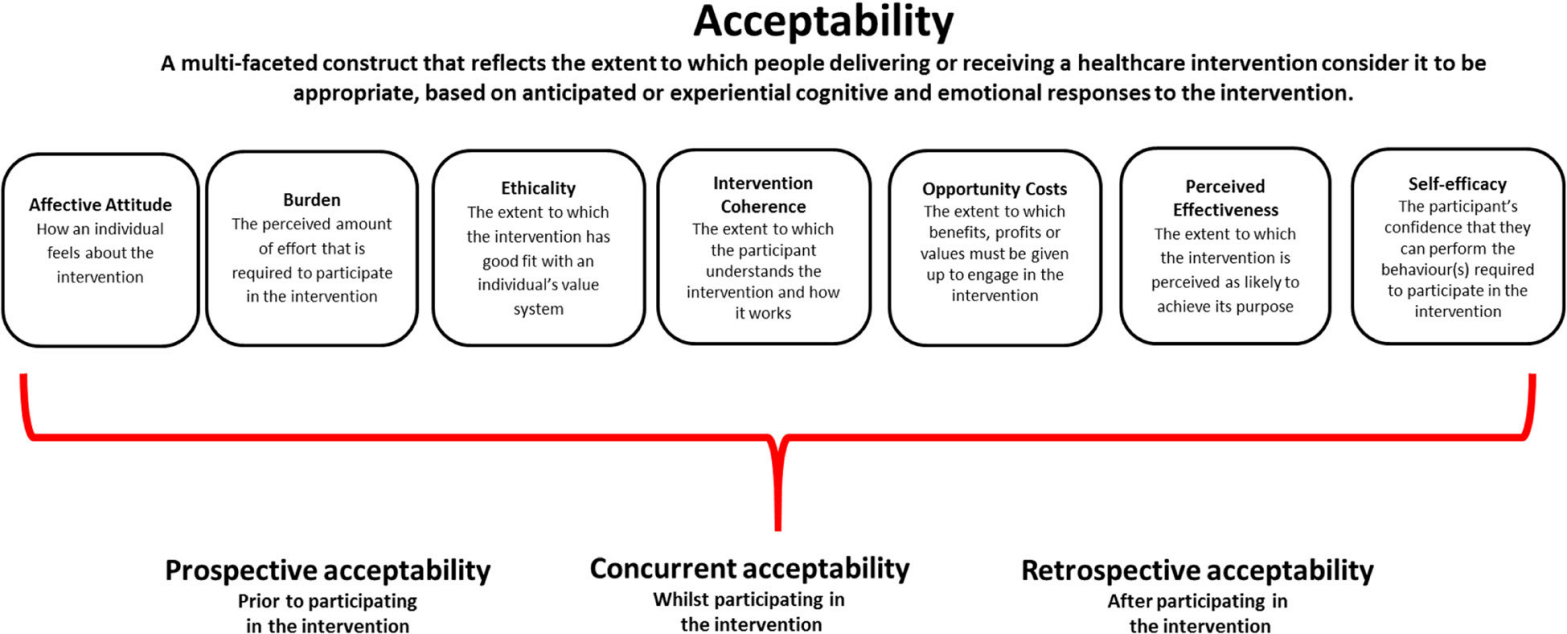
The theoretical framework of acceptability (v2) comprising seven component constructs. Note: The seven component constructs are presented alphabetically with their anticipated definitions. The extent to which they may cluster or influence each of the temporal assessments of acceptability is an empirical question

The process of identifying or writing explicit definitions for each of the proposed constructs in the theoretical framework of acceptability resulted in revisions to the TFA (v1) and the development of the revised TFA (v2) as we came to recognise inherent redundancy and overlap. Figure 3 presents the TFA (v2) comprising seven component constructs.

The inclusion of affective attitude as a construct in the TFA (v2) is in line with the findings of the overview of reviews, in which measures of attitude have been used to assess acceptability of healthcare interventions. Affective attitude is defined as “how an individual feels about taking part in an intervention”. The definition for burden was influenced by the Oxford dictionary definition, which defines burden as a “heavy load”. We define burden as “the perceived amount of effort that is required to participate in the intervention”. The TFA construct of burden focuses on the burden associated with participating in the intervention (e.g. participation requires too much time or expense, or too much cognitive effort, indicating the burden is too great) rather than the individual’s confidence in engaging in the intervention (see definition of self–efficacy below).

Opportunity costs are defined as “the extent to which benefits, profits, or values must be given up to engage in an intervention”, taken from the health economics literature. We changed the construct label of “ethical consequences” to “ethicality”, based on the Oxford dictionary definition of ethical, defined as “morally good or correct”. In the TFA (v2) ethicality is defined as “the extent to which the intervention has good fit with an individual’s value system”.

On reviewing the control items within the Illness Perception Questionnaire –Revised (IPQ-R), we realised all items focus on an individual’s perceived control of the illness for example, “there is a lot I can do to control my symptoms” ([56], p. 5). These items did not reflect the construct of personal control as we intended. We therefore considered how the relationship between confidence and personal control has been defined. Within the psychology literature the construct of self-efficacy has been defined in relation to confidence. Numerous authors have proposed that self-efficacy reflects confidence in the ability to exert control over one's own motivation, behaviour, and social environment [57]. We therefore considered a body of literature that groups control constructs together [38]. Self-efficacy is often operationalised as an individual’s confidence in his or her capability of performing a behaviour [58, 59]. In TFA (v2) we define the construct as “the participant’s confidence that they can perform the behaviour(s) required to participate in the intervention”.

The construct “intention” was removed from TFA (v2). This decision was taken upon a review of the extracted definitions of intention against our conceptual definition of acceptability. The Theory of Planned Behaviour [37] definition of intention states, “Intentions are assumed to capture the motivational factors that influence a behaviour; they are indications of how hard people are willing to try, of how much of an effort they are planning to exert, in order to perform the behaviour” ([37], p. 181). We propose that all other constructs within the TFA (v2) could be predictors of intention (e.g. willingness to participate in an intervention). If acceptability (assessed by measuring the component constructs in the TFA) is proposed to be a predictor of intention (to engage in the intervention), to avoid circularity it is important to retain a distinction between acceptability and intention.

We reviewed the definitions of the component constructs in TFA (v2) against our conceptual definition of acceptability to consider whether we were overlooking any important constructs that could further enhance the framework of acceptability. Drawing on our knowledge of health psychology theory we discussed how perceptions of acceptability may be influenced by participants’ and healthcare professionals’ understanding of a healthcare intervention and how it works in relation to the problem it targets. As a result, we propose an additional construct that we labelled “intervention coherence”. Our definition for this construct was informed by reviewing the illness perceptions literature. Moss-Morris et al., defined “illness coherence” as “the extent to which a patient’s illness representation provided a coherent understanding of the illness” (p. 2 [56]). Applying this definition within the TFA (v2), the construct of intervention coherence reflects an individual’s understanding of the perceived level of ‘fit’ between the components of the intervention and the intended aim of the intervention. We define intervention coherence as “the extent to which the participant understands the intervention, and how the intervention works”. Intervention coherence thus represents the face validity of the intervention to the recipient or deliverer.

Next we considered the applicability and relevance of the construct label “experience” for inclusion in the TFA (v2). Four of the constructs (affective attitude, burden, opportunity costs and perceived effectiveness) could include a definition that referred to acceptability of the intervention as experienced (Additional file 6) (e.g. opportunity costs- the benefits, profits, or values that were given up to engage in the intervention) as well as a definition that referred to the intervention as anticipated (as defined above). In TFA (v1) ‘experience’ was being used to distinguish between components of acceptability measured pre- or post-exposure to the intervention. In this sense experience is best understood as a characteristic of the assessment context rather than a distinct construct in its own right. We therefore did not include ‘experience’ as a separate construct in the TFA (v2). However, the distinction between anticipated and experienced acceptability is a key feature of the TFA (v2). We propose that acceptability can be assessed from two temporal perspectives (i.e. prospective/ forward-looking; retrospective / backward-looking) and at three different time points in relation to the intervention delivery period. The time points are (1) pre-intervention delivery (i.e. prior to any exposure to the intervention), (2) during intervention delivery (i.e. concurrent assessment of acceptability; when there has been some degree of exposure to the intervention and further exposure is planned), and (3) post-intervention delivery (i.e. following completion of the intervention or at the end of the intervention delivery period when no further exposure is planned). This feature of the TFA is in line with the findings of the overview of reviews in which review authors had described the time at which acceptability was assessed as pre–intervention, during the intervention and post-intervention.

## Discussion

We have presented the development of a theoretical framework of acceptability that can be used to guide the assessment of acceptability from the perspectives of intervention deliverers and recipients, prospectively and retrospectively. We propose that acceptability is a multi-faceted construct, represented by seven component constructs: affective attitude, burden, perceived effectiveness, ethicality, intervention coherence, opportunity costs, and self-efficacy.

### Overview of reviews

To our knowledge, this overview represents the first systematic approach to identifying how the acceptability of healthcare interventions has been defined, theorised and assessed. Most definitions offered within the systematic reviews focused on operational definitions of acceptability. For instance, number of dropouts, treatment discontinuation and other measurable variables such as side effects, satisfaction and uptake rates were used to infer the review authors’ definitions of acceptability. Measures applied in the reviews were mainly measures of observed behaviour. Whilst the use of measures of observed behaviour does give an indication of how many participants initially agree to participate in a trial versus how many actually complete the intervention, often reasons for discontinuation or withdrawal are not reported. There are several reasons why patients withdraw their participation that may or may not be associated with acceptability of the intervention. For example, a participant may believe the intervention itself is acceptable, however they may disengage with the intervention if they believe that the treatment has sufficiently ameliorated or cured their condition and is no longer required.

In the overview, only eight of 43 reviews combined observed behavioural and self-report measures in their assessments of acceptability. A combination of self–report measures and observed behaviour measures applied together may provide a clearer evaluation of intervention acceptability.

The overview shows that acceptability has sometimes been confounded with the construct of satisfaction. This is evident from the reviews that claim to have assessed acceptability using measures of satisfaction. However, while satisfaction with a treatment or intervention can only be assessed retrospectively, acceptability of a treatment or intervention can be assessed either prospectively or retrospectively. We therefore propose that acceptability is different to satisfaction as individuals can report (anticipated) acceptability prior to engaging in an intervention. We argue that acceptability can be and should be assessed prior to engaging in an intervention.

There is evidence that acceptability can be assessed prior to engaging in an intervention [14]. Sidani and colleagues [14] propose that there are several factors that can influence participants’ perceptions of the acceptability of the intervention prior to participating in the intervention, which they refer to as treatment acceptability. Factors such as participants’ attitudes towards the intervention, appropriateness, suitability, convenience and perceived effectiveness of the intervention have been considered as indicators of treatment acceptability.

### Theoretical framework of acceptability

The overview of reviews revealed no evidence of the development or application of theory as the basis for either operational or conceptual definitions of acceptability. This is surprising given that acceptability is not simply an attribute of an intervention but is rather a subjective evaluation made by individuals who experience (or expect to experience) or deliver (or expect to deliver) an intervention. The results of the overview highlight the need for a clear, consensual definition of acceptability. We therefore sought to theorise the concept of acceptability in order to understand what acceptability is (or is proposed to be) and what its components are (or are proposed to be).

The distinction between prospective and retrospective acceptability is a key feature of the TFA, and reflective of the overview of review results, which showed that acceptability has been assessed, before, during and after intervention delivery. We contend that prior to experiencing an intervention both patients and healthcare professionals can form judgements about whether they expect the intervention to be acceptable or unacceptable. These judgements may be based on the information provided about the intervention, or other factors outlined by Sidani et al., [14] in their conceptualisation of treatment acceptability. Assessment of anticipated acceptability prior to participation can highlight which aspects of the intervention could be modified to increase acceptability, and thus participation.

Researchers need to be clear about the purpose of acceptability assessments at different time points (i.e. pre-, during or post-intervention) and the stated purpose should be aligned to the temporal perspective adopted (i.e. prospective or retrospective acceptability). For example, when evaluating acceptability during the intervention delivery period (i.e. concurrent assessment) researchers have the option of assessing the experienced acceptability up to this point in time or assessing the anticipated acceptability in the future. Different temporal perspectives change the purpose of the acceptability assessment and may change the evaluation, e.g. when assessed during the intervention delivery period an intervention that is initially difficult to adjust to may have low experienced acceptability but high anticipated acceptability. Similarly post-intervention assessments of acceptability may focus on experienced acceptability based on participants’ experience of the intervention from initiation through to completion, or on anticipated acceptability based on participants’ views of what it would be like to continue with the intervention on an on-going basis .(e.g. as part of routine care). These issues are outside the scope of this article but we will elaborate further in a separate publication presenting our measures of the TFA (v2) constructs.

### Limitations

Although we have aimed to be systematic throughout the process, certain limitations should be acknowledged. The overview of reviews included systematic review papers that claimed to assess the acceptability of an intervention. It is possible that some papers were not identified by the search strategy as some restrictions were put in place to make the overview feasible. Nonetheless, the overview does provide a useful synthesis of how acceptability of healthcare interventions has been defined, assessed and theorised in systematic reviews of the effectiveness of healthcare interventions. In particular, the review highlights a distinct need to advance acceptability research.

A key objective of this paper was to describe the procedures by which the TFA were developed. Often methods applied to theorising are not clearly articulated or reported within literature [31]. We have been transparent in reporting the methods we applied to develop the TFA. Our work in theorising the concept of acceptability follows the process outlined by Hox [33]. However, the theorising process was also iterative as we continuously reviewed the results from the overview of reviews when making revisions from TFA (v1) to TFA (v2). We carefully considered the constructs in both TFA (v1) and TFA (v2) and how they represented our conceptual definition of acceptability. We also relied on and applied our own knowledge of health psychology theories in order to define the constructs. Given the large number of theories and models that contain an even larger number of constructs that are potentially relevant to acceptability this deductive process should be viewed as inevitably selective and therefore open to bias.

### Implications: The use of the TFA

We propose the TFA will be helpful in assessing the acceptability of healthcare interventions within the development, piloting and feasibility, outcome and process evaluation and implementation phases described by the MRC guidance on complex interventions [1, 12]. Table 3 outlines how the TFA can be applied qualitatively and quantitatively to assess acceptability in the different stages of the MRC intervention development and evaluation cycle.

**Table 3:** Proposed TFA methods applicable to the full complex intervention development and evaluation cycle

Development phase	Pilot and feasibility phase (before going to full scale trial)	Evaluation phase (trial context)	Implementation phase (scalability)
Qualitative	Qualitative	Qualitative	Qualitative
E.g. Semi-structured interviews or focus groups based on the TFA constructs with stakeholders to help guide decisions about the form, content and delivery mode of the proposed intervention components.	E.g. Semi-structured interviews or focus groups based on the TFA constructs with potential intervention recipients and deliverers. These should focus on the anticipated acceptability of content and mode of delivery of the intervention. Analysis may reveal aspects of intervention to modify.	E.g. Semi-structured interviews or focus groups on the TFA constructs with intervention recipients and deliverers about anticipated and/ or experienced acceptability. For a longitudinal analysis acceptability semi-structured interviews or focus groups should be conducted pre-intervention, during the intervention delivery period (concurrent) and post- intervention. E.g. Reflective diary entries, applying the TFA construct labels for experienced acceptability to guide participant diary entries.	E.g. Semi-structured interviews or focus groups based on the TFA constructs to assess experienced acceptability of the intervention/ service for recipients and deliverers. E.g. Reflective diary entries, applying the TFA construct labels for experienced acceptability to guide participant diary entries
Quantitative	Quantitative	Quantitative	Quantitative
E.g. Questionnaires or visual analogue rating scales based on the TFA constructs to assess anticipated acceptability amongst potential intervention deliverers or recipients.	E.g. Questionnaires or visual analogue rating scales based on the TFA constructs to assess anticipated acceptability amongst potential intervention deliverers or recipients. These measures should focus on the anticipated acceptability of content and mode of delivery of the intervention. Analysis may reveal aspects of intervention to modify.	E.g. Questionnaires or visual analogue rating scales based on the TFA constructs to assess experienced and/ or anticipated acceptability for intervention recipients and deliverers. For a longitudinal analysis acceptability measures should be administered pre-intervention, during the intervention delivery period (concurrent) and post- intervention.	E.g. Questionnaires or visual analogue rating scales on the TFA constructs to assess the experienced acceptability of the intervention/ service for recipients and deliverers.

The development phase of an intervention requires researchers to identify or develop a theory of change (e.g. what changes are expected and how they will be achieved) and to model processes and outcomes (e.g. using analogue studies and other evidence to identify the specific outcomes and appropriate measures) [1]. Explicit consideration of the acceptability of the intervention, facilitated by the TFA, at this stage would help intervention designers make informed decisions about the form, content and delivery mode of the proposed intervention components.

The MRC framework suggests that acceptability should be assessed in the feasibility phase [1]. The TFA will help intervention designers to operationalise this construct and guide the methods used to evaluate it, e.g. by adapting a generic TFA questionnaire or an interview schedule that we have developed (to be published separately). A pilot study often represents the first attempt to deliver the intervention and the TFA can be used at this stage to determine whether anticipated acceptability, for deliverers and recipients of the intervention, corresponds to their experienced acceptability. Necessary changes to aspects of the intervention (e.g. if recruitment was lower or attrition higher than expected) could be considered in light of experienced acceptability.

In the context of a definitive randomised controlled trial the TFA can be applied within a process evaluation to assess anticipated and experienced acceptability of the intervention to people receiving and/or delivering the healthcare intervention at different stages of intervention delivery. Findings may provide insights into reasons for low participant retention and implications for the fidelity of both delivery and receipt of the intervention [60]. High rates of participant dropout in trials may be associated with the burden of participating in research (e.g. filling out long follow–up questionnaires) and do not always reflect problems with acceptability of the intervention under investigation [61, 62]. Insights about acceptability from process evaluations may inform the interpretation of trial findings (e.g. where the primary outcomes were not as expected, a TFA assessment may indicate whether this is attributable to low acceptability leading to low engagement, or an ineffective intervention).

The TFA can also be applied to assess acceptability in the implementation phase when an intervention is scaled-up for wider rollout in ‘real world’ healthcare settings (e.g. patient engagement with a new service being offered as part of routine care).

## Conclusion

The acceptability of healthcare interventions to intervention deliverers and recipients is an important issue to consider in the development, evaluation and implementation phases of healthcare interventions. The theoretical framework of acceptability is innovative and provides conceptually distinct constructs that are proposed to capture key dimensions of acceptability. We have used the framework to develop quantitative (questionnaire items) and qualitative (topic guide) instruments for assessing the acceptability of complex interventions [63] (to be published separately). We offer the proposed multi-construct Theoretical Framework of Acceptability to healthcare researchers, to advance the science and practice of acceptability assessment for healthcare interventions.

## Additional files

Additional file 1: Systematic review filters. Description of data: List of each review filter applied to MEDLINE, Embase and PsycINFO. (DOCX 19 kb)
Additional file 2: References. Description of data: Citation details of all the systematic reviews included in the overview of reviews. (DOCX 40 kb)
Additional file 3: Behavioural assessments of acceptability. Description of data: How acceptability was assessed in the included systematic reviews based on measures of observed behaviour. (DOCX 12 kb)
Additional file 4: Self report assessments of acceptability. Description of data: Summary of the self-report measures of acceptability reported in the overview of reviews. (DOCX 12 kb)
Additional file 5: When was acceptability assessed?. Description of data: Summary of the timing of acceptability assessments relative to start of intervention reported in the papers identified in the systematic reviews. (DOCX 12 kb)
Additional file 6: Definition of TFA component constructs. Description of data: Definitions of each of the seven component constructs, including anticipated acceptability and experienced acceptability definitions for applicable constructs. (DOCX 13 kb)
