# Supplementary material for: Acceptability of healthcare interventions: an overview of reviews and development of a theoretical framework
Source: BMC Health Serv Res. 2017 Jan 26;17:88. doi: 10.1186/s12913-017-2031-8 (PMC5267473; doi:10.1186/s12913-017-2031-8)
Supplement: Additional file 1: — Systematic review filters. Description of data: List of each review filter applied to MEDLINE, Embase and PsycINFO. (DOCX 19 kb) [file 12913_2017_2031_MOESM1_ESM.docx]

**SYSTEMATIC REVIEW FILTERS**

**(adaptations of the SIGN Search Filters**

**http://www.sign.ac.uk/methodology/filters.html)**

**MEDLINE (Ovid)**

1. META-ANALYSIS/

2. META-ANALYSIS AS TOPIC/

3. (meta analy* or metaanaly*).tw.

4. REVIEW LITERATURE AS TOPIC/

5. (systematic* adj2 (review* or overview* or search*)).tw.

6. (literature adj2 (review or search*)).tw.

7. (medline or pubmed or cochrane or embase or cinahl or cinahl or lilacs or science citation index or "web of science" or conference proceedings or psyclit or psychlit or psycinfo or psychinfo).ab.

8. (search term* or published articles or search strateg*).ab.

9. (additional adj (papers or articles or sources)) .ab.

10. reference list*.ab.

11. (electronic adj (sources or resources or databases)).ab.

12. (bibliograph* or handsearch* or hand search* or manual* search*).ab.

13. (relevant adj (journals or articles)).ab.

14. or/1-13

15. Review.pt.

16. exp CLINICAL TRIALS AS TOPIC/

17. RANDOMIZED CONTROLLED TRIALS/

18. (data adj2 (extract* or analys*)).ab.

19. (selection criteria or critical appraisal).ab.

20. ((randomi* or controlled or cohort* or observational or retrospective* or nonrandomi* or case*) adj2 (trial* or stud*)).ab.

21. or/16-20

22. 15 and 21

23. 14 or 22

24. COMMENT/ or LETTER/ or EDITORIAL/

25. 23 not 24

26. ANIMALS/ not (ANIMALS/ and HUMANS/)

27. 25 not 26 (Watson-Jones et al., 2016)

Received

**Embase (Ovid)**
1. META ANALYSIS/

2. SYSTEMATIC REVIEW/

3. (meta analy* or metaanaly*).tw.
4. (systematic* adj2 (review* or overview* or search*)).tw.

5. (literature adj2 (review or search*)).tw.

6. (medline or pubmed or cochrane or embase or cinahl or cinahl or lilacs or science citation index or "web of science" or conference proceedings or or psyclit or psychlit or psycinfo or psychinfo).ab.

7. (search term* or published articles or search strateg*).ab.

8. (additional adj (papers or articles or sources)).ab.

9. reference list*.ab.

10. (electronic adj (sources or resources or databases)).ab.

11. (bibliograph* or handsearch* or hand search* or manual* search*).ab.

12. (relevant adj (journals or articles)).ab.

13. or/1-12

14. Review.pt.

15. (data adj2 (extract* or analys*)).ab.

16. (selection criteria or critical appraisal).ab.

17. ((randomi* or controlled or cohort* or observational or retrospective* or nonrandomi* or case*) adj2 (trial* or stud*)).ab.

18. or/15-17
19. 14 and 18
20. 13 or 19
21. (letter or editorial).pt.
22. 20 not 21

**PsycINFO (EBSCOhost)**
S1 DE META ANALYSIS

S2 MR SYSTEMATIC REVIEW

S3 MR META ANALYSIS
S4 TI ("meta analys*" OR metaanalys* OR "systematic review" OR "systematic overview" OR "systematic search*") OR AB ("meta analys*" OR metaanalys* OR "systematic review" OR "systematic overview" OR "systematic search*")
S5 TI ("literature review" OR "literature search*") OR AB ("literature review" OR "literature search*")
S6 AB (medline or pubmed or cochrane or embase or cinahl or cinahl or lilacs or "science citation index" or "web of science" or conference proceedings or or psyclit or psychlit or psycinfo or psychinfo)

S7. AB (search term* or published articles or search strategy* or reference list*)

S8 AB (additional N1 (papers or articles or sources))

S9 AB (electronic N1 (sources or resources or databases))

S10 AB (bibliograph* or handsearch* or hand search* or manual* search*)

S11 AB (relevant N1 (journals or articles))

S12 S1 OR S2 OR S3 OR S4 OR S5 OR S6 OR S7 OR S8 OR S9 OR S10 OR S11

S13. MR LITERATURE REVIEW

S14 AB (data N2 (extract* or analys*))

S15 AB (selection criteria or critical appraisal)

S16 TX ((randomi* or controlled or cohort* or observational or retrospective* or nonrandomi* or case*) N2 (trial* or stud*))

S17 S14 OR S15 OR S16

S18 S13 and S17

S19 S12 OR S18
