# Supplementary material for: Acceptability of healthcare interventions: an overview of reviews and development of a theoretical framework
Source: BMC Health Serv Res. 2017 Jan 26;17:88. doi: 10.1186/s12913-017-2031-8 (PMC5267473; doi:10.1186/s12913-017-2031-8)
Supplement: Additional file 2: — References. Description of data: Citation details of all the systematic reviews included in the overview of reviews. (DOCX 40 kb) [file 12913_2017_2031_MOESM2_ESM.docx]

**Citation details of all the systematic reviews included in the overview of reviews.**

1. Andrews, G., Cuijpers, P., Craske, M. G., McEvoy, P., & Titov, N. (2010). Computer therapy for the anxiety and depressive disorders is effective, acceptable and practical health care: a meta-analysis. *PLoS ONE, 5*(10), e13196.
2. Arrowsmith, M. E., Aicken, C. R., Saxena, S., & Majeed, A. (2012). Strategies for improving the acceptability and acceptance of the copper intrauterine device. *The Cochrane Library*.
3. Berlim, M., Van den Eynde, F., & Daskalakis, Z. J. (2013). A systematic review and meta-analysis on the efficacy and acceptability of bilateral repetitive transcranial magnetic stimulation (rTMS) for treating major depression. *Psychol Med, 43*(11), 2245-2254.
4. Berlim, M. T., Eynde, F., & Daskalakis, Z. J. (2013). EFFICACY AND ACCEPTABILITY OF HIGH FREQUENCY REPETITIVE TRANSCRANIAL MAGNETIC STIMULATION (rTMS) VERSUS ELECTROCONVULSIVE THERAPY (ECT) FOR MAJOR DEPRESSION: A SYSTEMATIC REVIEW AND META‐ANALYSIS OF RANDOMIZED TRIALS. *Depression and anxiety, 30*(7), 614-623
5. Berlim, M. T., McGirr, A., Van den Eynde, F., Fleck, M. P. A., & Giacobbe, P. (2014). Effectiveness and acceptability of deep brain stimulation (DBS) of the subgenual cingulate cortex for treatment-resistant depression: A systematic review and exploratory meta-analysis. *Journal of Affective Disorders, 159*, 31-38. doi: 10.1016/j.jad.2014.02.016
6. Berlim, M. T., Van den Eynde, F., & Daskalakis, Z. J. (2013). Clinically meaningful efficacy and acceptability of low-frequency repetitive transcranial magnetic stimulation (rTMS) for treating primary major depression: a meta-analysis of randomized, double-blind and sham-controlled trials. *Neuropsychopharmacology, 38*(4), 543-551.
7. Blenkinsopp, A., & Hassey, A. (2005). Effectiveness and acceptability of community pharmacy‐based interventions in type 2 diabetes: a critical review of intervention design, pharmacist and patient perspectives. *International Journal of Pharmacy Practice, 13*(4), 231-240.
8. Botella, C., Serrano, B., Baños, R. M., & Garcia-Palacios, A. (2015). Virtual reality exposure-based therapy for the treatment of post-traumatic stress disorder: A review of its efficacy, the adequacy of the treatment protocol, and its acceptability. *Neuropsychiatric Disease and Treatment, 11*.
9. Brooke-Sumner, C., Petersen, I., Asher, L., Mall, S., Egbe, C. O., & Lund, C. (2015). Systematic review of feasibility and acceptability of psychosocial interventions for schizophrenia in low and middle income countries. *BMC Psychiatry, 15*, 19.
10. Caldeira, D., Goncalves, N., Ferreira, J. J., Pinto, F. J., & Costa, J. (2015). Tolerability and Acceptability of Non-Vitamin K Antagonist Oral Anticoagulants in Atrial Fibrillation: Systematic Review and Meta-Analysis. *American Journal of Cardiovascular Drugs, 15*(4), 259-265.
11. Cipriani, A., Barbui, C., Salanti, G., Rendell, J., Brown, R., Stockton, S., . . . Geddes, J. R. (2011). Comparative efficacy and acceptability of antimanic drugs in acute mania: a multiple-treatments meta-analysis. *The Lancet, 378*(9799), 1306-1315.
12. Cipriani, A., Furukawa, T. A., Salanti, G., Geddes, J. R., Higgins, J. P. T., Churchill, R., . . . Barbui, C. (2009). Comparative efficacy and acceptability of 12 new-generation antidepressants: A multiple-treatments meta-analysis. *The Lancet, 373*, 746-758.
13. Davis, M. M., Freeman, M., Kaye, J., Vuckovic, N., & Buckley, D. I. (2014). A systematic review of clinician and staff views on the acceptability of incorporating remote monitoring technology into primary care. *Telemedicine Journal & E-Health, 20*(5), 428-438.
14. Diepeveen, S., Ling, T., Suhrcke, M., Roland, M., & Marteau, T. M. (2013). Public acceptability of government intervention to change health-related behaviours: a systematic review and narrative synthesis. *BMC Public Health, 13*(1), 756.
15. El-Den, S., O'Reilly, C. L., & Chen, T. F. (2015). A systematic review on the acceptability of perinatal depression screening. *Journal of Affective Disorders, 188*, 284-303.
16. Figueroa, C., Johnson, C., Verster, A., & Baggaley, R. (2015). Attitudes and acceptability on HIV self-testing among key populations: A literature review. *AIDS and Behavior, 19*(11), 1949-1965. doi: 10.1007/s10461-015-1097-8
17. Galdas, P., Darwin, Z., Kidd, L., Blickem, C., McPherson, K., Hunt, K., . . . Richardson, G. (2014). The accessibility and acceptability of self-management support interventions for men with long term conditions: a systematic review and meta-synthesis of qualitative studies. *BMC Public Health, 14*(1), 1.
18. Glenton, C., Khanna, R., Morgan, C., & Nilsen, E. S. (2013). The effects, safety and acceptability of compact, pre‐filled, autodisable injection devices when delivered by lay health workers. *Tropical Medicine & International Health, 18*(8), 1002-1016.

1. Gonzalez-Rodriguez, A., Catalan, R., Penades, R., Garcia-Rizo, C., Bioque, M., Parellada, E., & Bernardo, M. (2015). Profile of paliperidone palmitate once-monthly long-acting injectable in the management of schizophrenia: Long-term safety, efficacy, and patient acceptability - A review. *Patient Preference and Adherence, 9*, 695-706.
2. Häuser, W., Petzke, F., Üçeyler, N., & Sommer, C. (2011). Comparative efficacy and acceptability of amitriptyline, duloxetine and milnacipran in fibromyalgia syndrome: a systematic review with meta-analysis. *Rheumatology, 50*(3), 532-543.
3. Kaltenthaler, E., Sutcliffe, P., Parry, G., Rees, A., & Ferriter, M. (2008). The acceptability to patients of computerized cognitive behaviour therapy for depression: a systematic review. *Psychological Medicine, 38*, 1521-1530.
4. Kedge, E. M. (2009). A systematic review to investigate the effectiveness and acceptability of interventions for moist desquamation in radiotherapy patients. *Radiography, 15*, 247-257.
5. Koesters, M., Guaiana, G., Cipriani, A., Becker, T., & Barbui, C. (2013). Agomelatine efficacy and acceptability revisited: systematic review and meta-analysis of published and unpublished randomised trials. *The British Journal of Psychiatry, 203*(3), 179-187.
6. Kulier, R., Helmerhorst, F. M., Maitra, N., & Gülmezoglu, A. M. (2004). Effectiveness and acceptability of progestogens in combined oral contraceptives–a systematic review. *Reproductive health, 1*(1), 1.
7. Lewis, C., Pearce, J., & Bisson, J. I. (2012). Efficacy, cost-effectiveness and acceptability of self-help interventions for anxiety disorders: systematic review. *The British Journal of Psychiatry, 200*(1), 15-21.
8. Liddon, N., Hood, J., Wynn, B. A., & Markowitz, L. E. (2010). Acceptability of human papillomavirus vaccine for males: a review of the literature. *Journal of Adolescent Health, 46*(2), 113-123.
9. Linde, K., Kriston, L., Rucker, G., Jamil, S., Schumann, I., Meissner, K., . . . Schneider, A. (2015). Efficacy and acceptability of pharmacological treatments for depressive disorders in primary care: systematic review and network meta-analysis. *Annals of Family Medicine, 13*(1), 69-79.
10. Littlejohn, C. (2006). Does socio-economic status influence the acceptability of, attendance for, and outcome of, screening and brief interventions for alcohol misuse: A review. *Alcohol and Alcoholism, 41*, 540-545.
11. Liu, J., Dong, J., Wang, L., Su, Y., Yan, P., & Sun, S. (2013). Comparative efficacy and acceptability of antidepressants in Parkinson's disease: a network meta-analysis. *PLoS ONE, 8*(10), e76651.
12. Maddocks, M., Mockett, S., & Wilcock, A. (2009). Is exercise an acceptable and practical therapy for people with or cured of cancer? A systematic review. *Cancer Treatment Reviews, 35*, 383-390.
13. Maneeton, N., Maneeton, B., Eurviriyanukul, K., & Srisurapanont, M. (2013). Efficacy, tolerability, and acceptability of bupropion for major depressive disorder: a meta-analysis of randomized–controlled trials comparison with venlafaxine. *Drug design, development and therapy, 7*, 1053.
14. Marrazzo, J. M., & Scholes, D. (2008). Acceptability of urine-based screening for Chlamydia trachomatis in asymptomatic young men: a systematic review. *Sexually Transmitted Diseases, 35*, S28-33.
15. McClung, E., & Blumenthal, P. (2012). Efficacy, safety, acceptability and affordability of cryotherapy: a review of current literature. *Minerva ginecologica, 64*(2), 149-171.
16. Muftin, Z., & Thompson, A. R. (2013). A systematic review of self-help for disfigurement: Effectiveness, usability, and acceptability. *Body Image, 10*(4), 442-450.
17. Ostuzzi, G., Benda, L., Costa, E., & Barbui, C. (2015). Efficacy and acceptability of antidepressants on the continuum of depressive experiences in patients with cancer: Systematic review and meta-analysis. *Cancer Treatment Reviews, 41*(8), 714-724.
18. Peters, A., van Driel, F., & Jansen, W. (2014). Acceptability of the female condom by sub-Saharan African women: a literature review. *African Journal of Reproductive Health, 18*(4), 34-44.
19. Robinson, L., Hutchings, D., Dickinson, H. O., Corner, L., Beyer, F., Finch, T., . . . Bond, J. (2007). Effectiveness and acceptability of non-pharmacological interventions to reduce wandering in dementia: A systematic review. *International Journal of Geriatric Psychiatry, 22*, 9-22.
20. Rodriguez, M. I., & Gordon-Maclean, C. (2014). The safety, efficacy and acceptability of task sharing tubal sterilization to midlevel providers: a systematic review. *Contraception, 89*(6), 504-511.
21. Skapinakis, P., Bakola, E., Salanti, G., Lewis, G., Kyritsis, A. P., & Mavreas, V. Efficacy and acceptability of selective serotonin reuptake inhibitors for the treatment of depression in Parkinson's disease: A systematic review and meta-analysis of randomized controlled trials. *BMC Neurology, 10*.
22. Skapinakis, P., Bakola, E., Salanti, G., Lewis, G., Kyritsis, A. P., & Mavreas, V. (2010). Efficacy and acceptability of selective serotonin reuptake inhibitors for the treatment of depression in Parkinson's disease: a systematic review and meta-analysis of randomized controlled trials. *BMC Neurology, 10*(1), 1.
23. Tarrier, N., Liversidge, T., & Gregg, L. (2006). The acceptability and preference for the psychological treatment of PTSD. *Behaviour Research and Therapy, 44*(11), 1643-1656.
24. Van Lieshout, R. J., & MacQueen, G. M. (2010). Efficacy and acceptability of mood stabilisers in the treatment of acute bipolar depression: systematic review. *The British Journal of Psychiatry, 196*(4), 266-273.
25. Yang, Z., & Zhan, S. (2015). Comparative efficacy and acceptability of glycemic control of glucagons like peptide-1 receptor agonists for type 2 diabetes: A systematic review and network meta-analysis. *Journal of the American College of Cardiology, 1)*, C128.
