# Supplementary material for: Acceptability of healthcare interventions: an overview of reviews and development of a theoretical framework
Source: BMC Health Serv Res. 2017 Jan 26;17:88. doi: 10.1186/s12913-017-2031-8 (PMC5267473; doi:10.1186/s12913-017-2031-8)
Supplement: Additional file 3: — Behavioural assessments of acceptability. Description of data: How acceptability was assessed in the included systematic reviews based on measures of observed behaviour. (DOCX 12 kb) [file 12913_2017_2031_MOESM3_ESM.docx]

**Behavioural assessments of acceptability**

| Measures of observed behaviour | n |
| --- | --- |
| Drop –out rates | 10 |
| All cause discontinuation rates | 4 |
| Willingness to participate/ take test in future | 2 |
| Treatment discontinuation | 2 |
| Discontinuation and removal rate | 1 |
| Discontinuation, attrition, adherence, non-compliance | 1 |
| Rates of uptake, adherence and completion of exercise | 1 |
| Withdrawal rates | 1 |
| Uptake | 1 |
| Total | 23 |
