# Supplementary material for: Acceptability of healthcare interventions: an overview of reviews and development of a theoretical framework
Source: BMC Health Serv Res. 2017 Jan 26;17:88. doi: 10.1186/s12913-017-2031-8 (PMC5267473; doi:10.1186/s12913-017-2031-8)
Supplement: Additional file 4: — Self report assessments of acceptability. Description of data: Summary of the self-report measures of acceptability reported in the overview of reviews. (DOCX 12 kb) [file 12913_2017_2031_MOESM4_ESM.docx]

Self -report assessments of acceptability

| Self-report assessment | n |
| --- | --- |
| Satisfaction measures | 6 |
| Attitudes | 1 |
| Interviews on users perceptions, experiences and attitudes towards intervention | 1 |
| Surveys (hypothetical scenarios) | 1 |
| Open ended questions | 1 |
| Interviews (barriers and facilitators of access to intervention and support activities) | 1 |
| Side effects | 1 |
| Total | 12 |
