# Supplementary material for: Acceptability of healthcare interventions: an overview of reviews and development of a theoretical framework
Source: BMC Health Serv Res. 2017 Jan 26;17:88. doi: 10.1186/s12913-017-2031-8 (PMC5267473; doi:10.1186/s12913-017-2031-8)
Supplement: Additional file 5: — When was acceptability assessed?. Description of data: Summary of the timing of acceptability assessments relative to start of intervention reported in the papers identified in the systematic reviews. (DOCX 12 kb) [file 12913_2017_2031_MOESM5_ESM.docx]

When acceptability was assessed (relative to start of intervention) reported in systematic reviews.

| Assessment of acceptability (relative to start of intervention) | n |
| --- | --- |
| Pre intervention | 2 |
| During intervention | 7 |
| Post Intervention | 17 |
| Unclear | 3 |
| Not reported | 14 |
| Total | 43 |
