# Supplementary material for: Acceptability of healthcare interventions: an overview of reviews and development of a theoretical framework
Source: BMC Health Serv Res. 2017 Jan 26;17:88. doi: 10.1186/s12913-017-2031-8 (PMC5267473; doi:10.1186/s12913-017-2031-8)
Supplement: Additional file 6: — Definition of TFA component constructs. Description of data: Definitions of each of the seven component constructs, including anticipated acceptability and experienced acceptability definitions for applicable constructs. (DOCX 13 kb) [file 12913_2017_2031_MOESM6_ESM.docx]

Definitions of the component constructs in the Theoretical framework of acceptability

| Theoretical Framework of acceptability ( TFA) | Definition |
| --- | --- |
| Ethicality | The extent to which the intervention has good fit with an individual’s value system |
| Affective  Attitude | Anticipated Affective Attitude: How an individual feels about the intervention, prior to taking part  Experienced Affective Attitude: How an individual feels about the intervention, after taking part |
| Burden | Anticipated burden: The perceived amount of effort that is required to participate in the intervention  Experienced burden: the amount of effort that was required to participate in the intervention |
| Opportunity  Costs | Anticipated opportunity cost : The extent to which benefits, profits, or values must be given up to engage in the intervention  Experienced opportunity cost: the benefits, profits or values that were given up to engage in the intervention |
| Perceived  effectiveness | Anticipated effectiveness: the extent to which the intervention is perceived to be likely to achieve its purpose  Experienced effectiveness: the extent to which the intervention is perceived to have achieved its intended purpose |
| Self-efficacy | The participant's confidence that they can perform the behaviour(s) required to participate in the intervention |
| Intervention Coherence | The extent to which the participant understands the intervention and how it works |
